# Carrageenans from the Red Seaweed *Sarconema filiforme* Attenuate Symptoms of Diet-Induced Metabolic Syndrome in Rats

**DOI:** 10.3390/md18020097

**Published:** 2020-01-31

**Authors:** Ryan du Preez, Nicholas Paul, Peter Mouatt, Marwan E. Majzoub, Torsten Thomas, Sunil K. Panchal, Lindsay Brown

**Affiliations:** 1 Functional Foods Research Group, University of Southern Queensland, Toowoomba, QLD 4350, Australia; r.dupreez@cqu.edu.au (R.d.P.); S.Panchal@westernsydney.edu.au (S.K.P.); 2 School of Health and Wellbeing, University of Southern Queensland, Toowoomba, QLD 4350, Australia; 3 School of Science and Engineering, University of the Sunshine Coast, Maroochydore, QLD 4558, Australia; npaul@usc.edu.au; 4 Southern Cross Plant Science, Southern Cross University, Lismore, NSW 2480, Australia; Peter.Mouatt@scu.edu.au; 5 Centre for Marine Science and Innovation, University of New South Wales, Sydney, NSW 2052, Australia; m.majzoub@unsw.edu.au (M.E.M.); t.thomas@unsw.edu.au (T.T.); 6 School of Biological, Earth and Environmental Sciences, University of New South Wales, Sydney, NSW 2052, Australia

**Keywords:** algae, *Sarconema filiforme*, sulfated polysaccharides, *ι*-carrageenan, prebiotics, gut microbiota, aquaculture, nutraceutical

## Abstract

Carrageenans are thickening and gelling agents that may provide health benefits. Iota (ι)-carrageenan, a linear sulfated polysaccharide, is produced by the red seaweed, *Sarconema filiforme*. This study investigated the potential of this seaweed as a functional food for the reversal of metabolic syndrome and possible mechanisms. Male Wistar rats were divided into four groups in a 16-week protocol: corn starch diet-fed rats (C); C rats supplemented with 5% *S. filiforme* for the last 8 weeks (CSF); high-carbohydrate, high-fat diet-fed rats (H); and H rats supplemented with 5% *S. filiforme* for the last 8 weeks (HSF). *S. filiforme* was produced in tank-based aquaculture yielding 27 g dry weight/day/m^2^ of culture area. H rats developed obesity, hypertension, dyslipidaemia, glucose intolerance, fatty liver and increased left ventricular collagen deposition. *S. filiforme* supplementation decreased body weight, abdominal and liver fat, systolic blood pressure, plasma total cholesterol concentrations, and plasma activities of alanine transaminase and aspartate transaminase. *S. filiforme* supplementation modulated gut microbiota without changing the Firmicutes to Bacteroidetes ratio. *S. filiforme* improved symptoms of high-carbohydrate, high-fat diet-induced metabolic syndrome in rats. Possible mechanisms include a reduced infiltration of inflammatory cells into organs as well as prebiotic actions in the gastrointestinal tract.

## 1. Introduction

Carrageenans are a group of high molecular weight (>100 kDa) sulfated polygalactans isolated from red seaweeds (Rhodophyceae) that are “generally regarded as safe” for routine use as gelling and thickening agents in foods [1]. The three types of carrageenans, named kappa (κ), iota (ι) and lambda (λ), have one, two and three sulfate groups per disaccharide unit, respectively [1]. The major commercially cultivated warm-water species for carrageenans are *Kappaphycus alvarezii* and *Eucheuma denticulatum* [2], producing κ- and ι-carrageenans, respectively. These seaweeds are grown on a commercial scale primarily in Indonesia, the Philippines, Malaysia, Brazil and Tanzania [3]. *Sarconema filiforme* is a red seaweed containing ι-carrageenan, distributed throughout the tropical and subtropical Indo-Pacific, including along the eastern and western coasts of Australia [4,5].

The health benefits of red seaweeds include obesity reduction, decreased lipid absorption and modification of the binding of cholesterol in the gastrointestinal tract thereby reducing cardiovascular disease risk [1,6]. However, there have been no studies on *Sarconema* species or their major constituents in metabolic syndrome for reduction of obesity, hypertension, fatty liver, hyperlipidaemia or diabetes. Recently, 0.12% ι-carrageenan has been used in a nasal spray in patients against rhinitis, demonstrating safety and efficacy [7]. In our previous study, rats fed with κ-carrageenan-producing *K. alvarezii* showed normalised body weight and adiposity, lowered systolic blood pressure, improved heart and liver structure, and lowered plasma lipids, compared to diet-induced obese rats [8].

The aim of the present study was to determine changes in parameters defining cardiovascular and metabolic health following chronic consumption of *S. filiforme*, produced through intensive tank-based cultivation, as a potential commercial source of ι-carrageenan. Firstly, we evaluated the proximate and elemental composition of *S. filiforme*. Secondly, a validated diet-induced rat model of metabolic syndrome that closely mimics the symptoms of human metabolic syndrome was used to measure cardiovascular and metabolic parameters. We measured systolic blood pressure, diastolic stiffness, cardiac inflammatory cells and collagen deposition in the heart for cardiovascular effects; plasma liver enzyme activities, inflammatory cells and fat vacuoles in the liver for liver effects; and body weight, total cholesterol and triglyceride concentrations, and glucose and insulin tolerance tests for metabolic effects. Further, we characterised the microbial composition of faecal samples after seaweed treatment, since functional foods may reverse obesity-induced cardiometabolic changes through alterations in the gut microbiota [9]. We hypothesised that 5% *S. filiforme* supplementation for the last 8 weeks will reverse the changes induced by the high-carbohydrate, high-fat diet to a greater extent than *K. alvarezii* due to the additional sulfate group in the disaccharide units of ι-carrageenan. The mechanisms of these effects could include the actions of ι-carrageenan as a prebiotic in the colon and to prevent infiltration of inflammatory cells into organs such as the heart and liver.

## 2. Results

### 2.1. Preparation of *S. filiforme* Powder and Analysis

**Figure 1:**
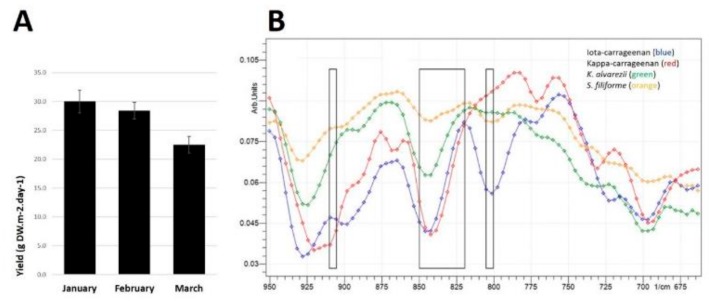
(**A**) Biomass yields of *Sarconema filiforme* between January and March 2018. Data show means ± SEM, n = 20–27 weekly growth measurements from 1000 L outdoor tanks from each month. (**B**) Attenuated Total Reflectance-Fourier-Transform Infrared Spectroscopy (ATR-FTIR) transmittance from 950 to 675 cm^−1^ of ι-carrageenan (blue line), κ-carrageenan (red line), *Kappaphycus alvarezii* (green line) and *Sarconema filiforme* (orange line). Far left rectangle showing 900–905 cm^−1^, middle rectangle showing 820-850 cm^−1^ and far right rectange showing 800–805 cm^−1^.

*S. filiforme* samples were taken from batches grown in the Austral summer during January, February and March 2018 with an overall average yield of 27 g dry weight/day/m^2^ (Figure 1A). This equates to weekly production of 189 g dry weight/m^2^ of available culture area for intensive land-based production of the seaweed. In the current study, high-carbohydrate, high-fat diet-fed rats treated with *S. filiforme* (HSF) consumed 5% *S. filiforme* for the last eight weeks (~1.05 g/day). Using the Reagan-Shaw calculation for rat-to-human scaling, this equates to approximately 6.3 g dry weight of seaweed/day for humans [10]. For perspective, based on the overall average yield and with 42 m^2^ of culture area, the University of the Sunshine Coast facility at Bribie Island could provide continuous seaweed at this dose for 179 people; at this overall yield, 5.7 km^2^ of culture area could provide continuous seaweed for the current Australian population. The seaweed powder contained (in % dry weight): 34.4% carbohydrates (comprising 21.7% total dietary fibre, including 9.6% insoluble fibre and 12.2% of soluble fibre, almost all as ι-carrageenan), 11.8% protein, 1.4% lipid, 50.8% ash and 1.6% moisture (Table 1). Elemental analysis showed 21.2% C, 3.3% H, 2.6% N, 4.2% S, 18.9% K and 2.7% Na (Table 1). The energy content was 8.7 kJ/g.

### 2.2. ATR-FTIR of *S. filiforme* and *K. alvarezii*

The normalised transmission spectra of reference samples of ι-carrageenan and κ-carrageenan are given with those of *S. filiforme* and *K. alvarezii* (Figure 1B). The peak at approximately 800–805 cm^−1^ is most prominent in ι-carrageenan, which is indicative of 3,6-anhydro-d-galactose 2-sulfate and the bands between 820 and 850 cm^−1^ are indicative of galactose 2-sulfate, galactose 4-sulfate and galactose 2,6-disulfate, which are characteristic of carrageenans. The band at ~800–805 cm^−1^ is more prominent in ι-carrageenan and *S. filiforme* samples. The band at ~905–910 cm^−1^ is indicative of anhydro-galactose-2-sulfate which is consistent between ι-carrageenan and *S. filiforme* samples.

### 2.3. Physiological and Metabolic Responses

**Figure 2:**
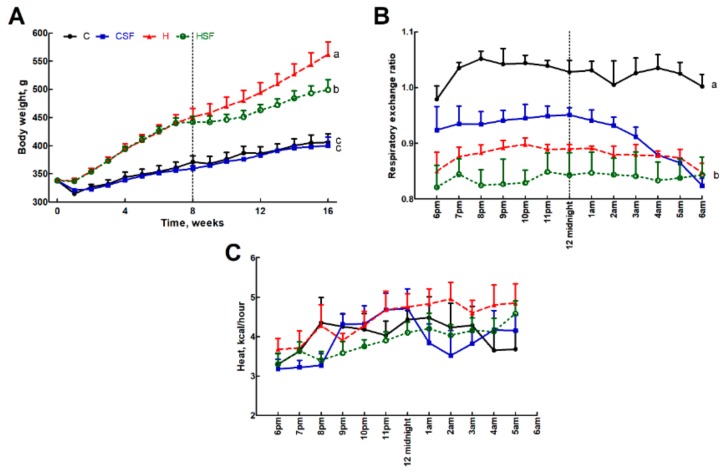
(**A**) Body weight, (**B**) 12-hour indirect calorimeter data for respiratory exchange ratio and (**C**) heat production in corn starch diet-fed rats (C), corn starch diet-fed rats supplemented with *Sarconema filiforme* (CSF), high-carbohydrate, high-fat diet-fed rats (H) and high-carbohydrate, high-fat diet-fed rats supplemented with *Sarconema filiforme* (HSF). End-point means with unlike superscripts differ (a, b or c), *p* < 0.05.

Food intake was higher in corn starch diet-fed rats (C) compared to high-carbohydrate, high-fat diet-fed rats (H). HSF had lower food intake than H rats (Table 2). The body weight of H rats was greater than C rats and that of HSF rats was lower than H rats (Figure 2A). Lean mass was unchanged in all groups. Fat mass measurements were consistent with body weight measurements (Table 2).

C rats had higher respiratory exchange ratio compared to H rats, while corn starch diet-fed rats treated with *S. filiforme* (CSF) and HSF were the same as H rats (Figure 2B). C rats had lower heat production than H rats, while CSF was higher than C and HSF was lower than H (Figure 2C). Total abdominal fat was highest in H rats followed by HSF, CSF and C rats.

**Figure 3:**
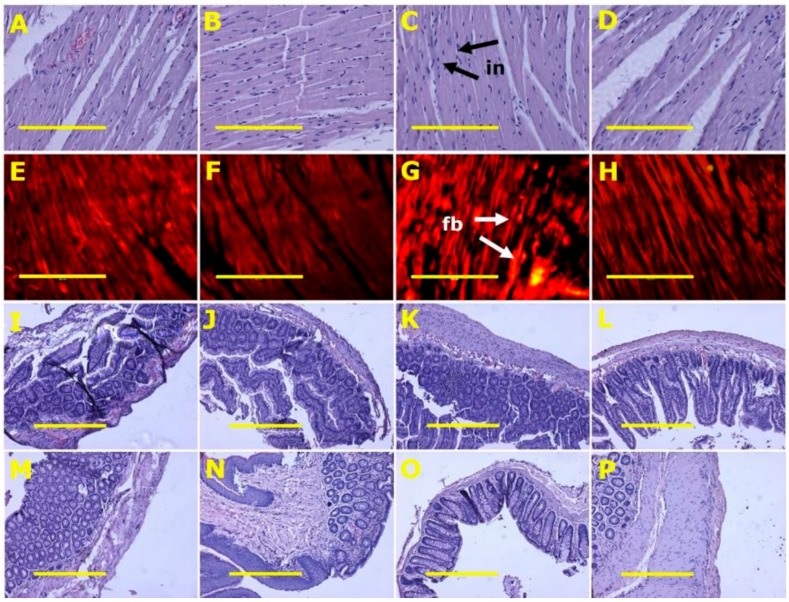
Heart inflammation (**A**–**D**) using haematoxylin and eosin stain; heart fibrosis (**E**–**H**) using picrosirius red stain; ileum (**I**–**L**) and colon (**M**–**P**) structure using haematoxylin and eosin stain in corn starch diet-fed rats (**A**,**E**,**I**,**M**), corn starch diet-fed rats supplemented with *Sarconema filiforme* (**B**,**F**,**J**,**N**), high-carbohydrate, high-fat diet-fed rats (**C**,**G**,**K**,**O**) and high-carbohydrate, high-fat diet-fed rats supplemented with *Sarconema filiforme* (**D**,**H**,**L**,**P**). Fibrosis = fb; inflammation = in. Scale bar for images A-H is 200µm (20×) and for images I-P is 100µm (10×).

After eight weeks, systolic blood pressures of H diet-fed groups (H and HSF) were higher than C diet-fed groups (C and CSF). Systolic blood pressures in H rats were higher at 16 weeks than in C rats. HSF rats had decreased systolic blood pressures compared to H control rats. Diastolic stiffness was higher in H rats compared to C rats. HSF rats showed normalised diastolic stiffness. Left ventricular with septum wet weights and right ventricular wet weights were unchanged across all groups. Left ventricles from H rats showed increased infiltration of inflammatory cells and collagen deposition, whereas these changes were not seen in left ventricles from C rats (Table 2). Infiltration of inflammatory cells and collagen deposition was normal in hearts from CSF rats and decreased in HSF rats (Figure 3 and Table 2).

Plasma triglyceride concentrations were higher in H and HSF rats compared to C and CSF rats; plasma total cholesterol concentrations were highest in H rats and lowest in C, CSF and HSF rats; and plasma non-esterified fatty acids were unchanged across all groups (Table 2). C rats had lower basal blood glucose concentrations compared to H rats. Intervention with *S. filiforme* reduced basal blood glucose concentrations. The blood glucose area under the curve was not different between groups (Table 2).

**Figure 4:**
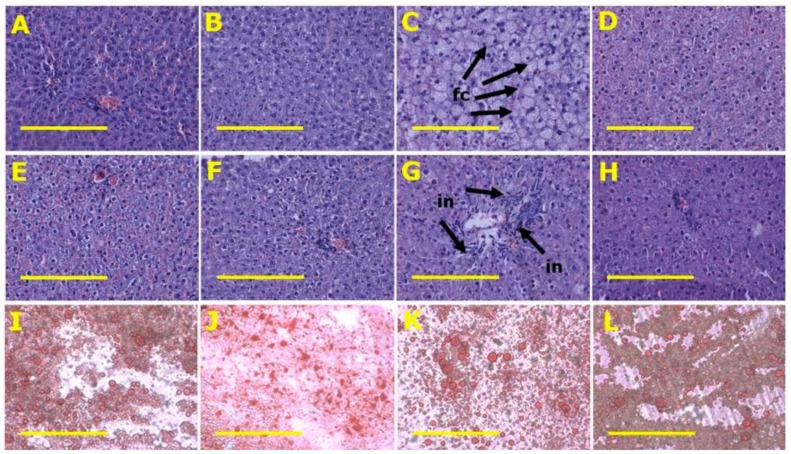
Fat vacuoles (**A**–**D**) and inflammation (**E**–**H**) using haematoxylin and eosin and liver fat using oil red O stain (**I**–**L**) in corn starch diet-fed rats (**A**,**E**,**I**), corn starch diet-fed rats supplemented with *Sarconema filiforme* (**B**,**F**,**J**), high-carbohydrate, high-fat diet-fed rats (**C**,**G**,**K**) and high-carbohydrate, high-fat diet-fed rats supplemented with *Sarconema filiforme* (**D**,**H**,**L**). Fat vacuole containing cells = fc; inflammatory cells = in. Scale bar is 200µm (20×).

H rats had higher plasma activities of ALT and AST compared to C rats. CSF and HSF rats were the same as C rats (Table 2). Livers from H rats showed increased fat deposition and infiltration of inflammatory cells compared to livers from C rats (Table 2 and Figure 4). HSF rats had reduced fat deposition compared to H rats (Figure 4).

### 2.4. Gut Structure and Microbiota

Histology of ileum and colon did not show any structural abnormalities in the experimental groups with normal crypt depth, villi length and goblet cells, and less inflammatory cell infiltration (Figure 3).

The gut microbiota was characterised by a total of 739,069 quality-filtered sequences that were clustered into 1233 zero-radius operational taxonomic units (zOTUs), which are roughly equivalent to the taxonomic level of species or strains. The calculated rarefaction curves based on rarefied and unrarefied data as well as Good’s coverage of 99.67 ± 0.10% showed that the bacterial communities were almost fully recovered by the surveying effort.

**Figure 5:**
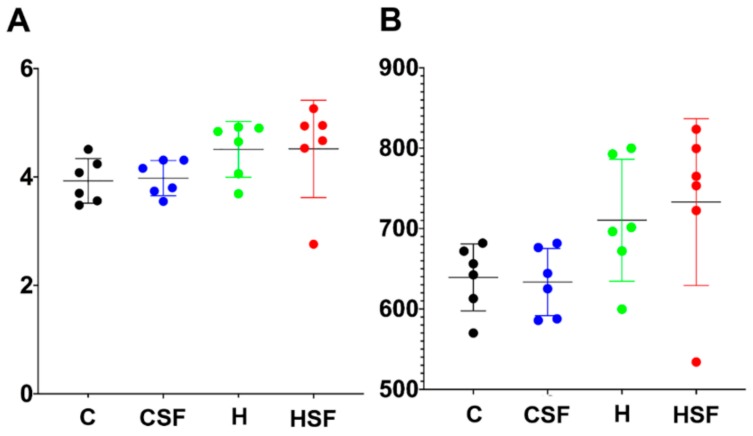
(**A**) Shannon diversity and (**B**) richness of faecal samples. C, corn starch diet-fed rats; CSF, corn starch diet-fed rats supplemented with *Sarconema filiforme*; H, high-carbohydrate, high-fat diet-fed rats; HSF, high-carbohydrate, high-fat diet-fed rats supplemented with *Sarconema filiforme*.

**Figure 6:**
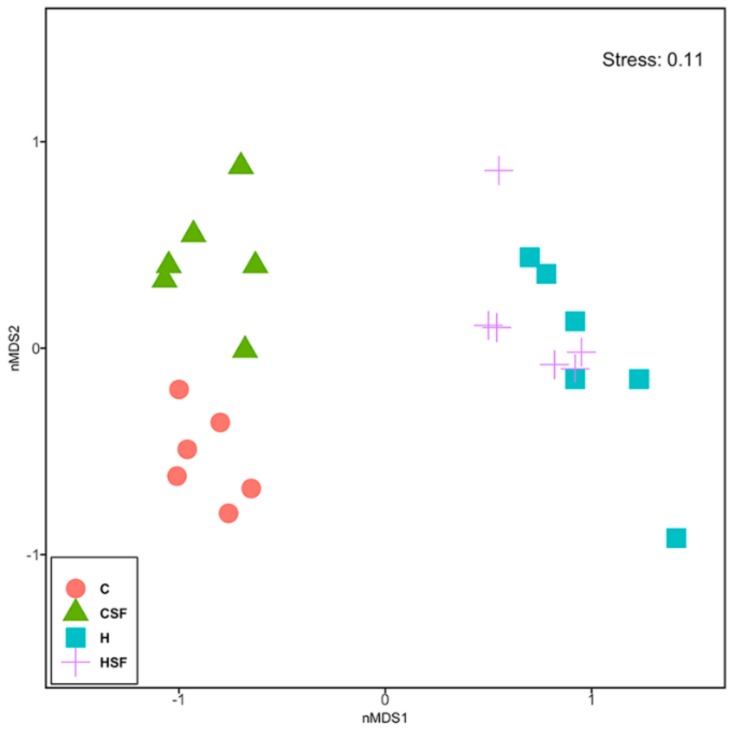
Multi-disciplinary scaling (MDS) plot of bacterial community structure of faecal samples from different feeding regimes. C, corn starch diet-fed rats; CSF, corn starch diet-fed rats supplemented with *Sarconema filiforme*; H, high-carbohydrate, high-fat diet-fed rats; HSF, high-carbohydrate, high-fat diet-fed rats supplemented with *Sarconema filiforme*.

There was no difference in Shannon’s diversity or richness between the four groups (Figure 5). Diet and seaweed supplement both affected the overall bacterial community structure based on Bray-Curtis dissimilarity (Figure 6, Table 3; PERMANOVA, both *p* = 0.0001), and there was an interaction between the two factors (Figure 6, Table 3; PERMANOVA, *p* = 0.003). There were pairwise differences between the C and H groups indicating an effect of basal feed on the bacterial community structure (*p* = 0.0017). The addition of *S. filiforme* changed the bacterial communities (CSF, *p* = 0.0014; HSF, *p* = 0.0396). Bacterial communities in the CSF group were more variable compared to the C group (Figure 6, Table 3; PERMDISP; *p* = 0.022).

**Figure 7:**
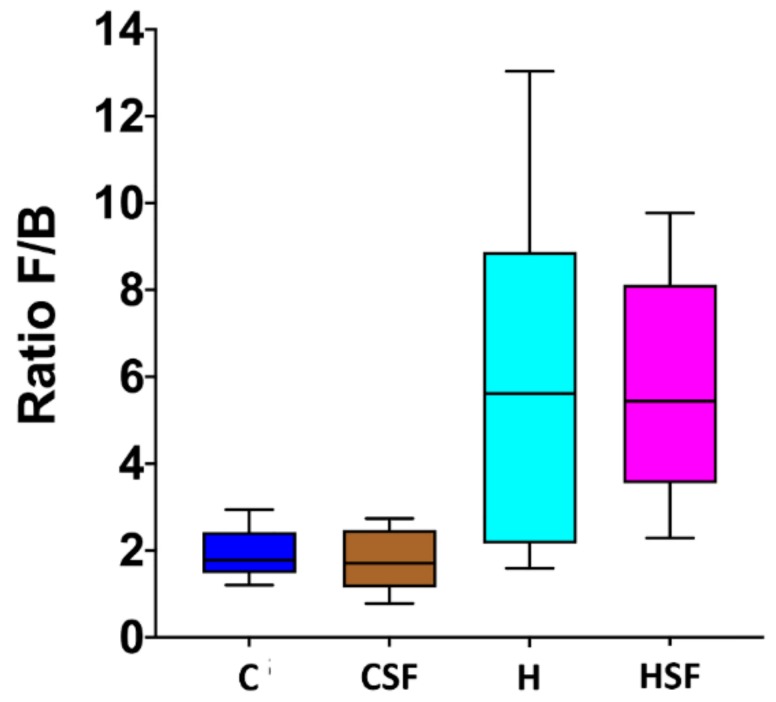
Effect of supplementation of diet (C or H) with *Sarconema filiforme* on the ratio of Firmicutes and Bacteroidetes abundances in rat faecal samples. Statistical analysis performed using ANOVA with Tukey’s post hoc test for multiple comparisons. C, corn starch diet-fed rats; CSF, corn starch diet-fed rats supplemented with *Sarconema filiforme*; H, high-carbohydrate, high-fat diet-fed rats; HSF, high-carbohydrate, high-fat diet-fed rats supplemented with *Sarconema filiforme*.

C rats and CSF rats had lower ratios of Firmicutes to Bacteroidetes (F/B ratio) compared to H and HSF rats (Figure 7). There was no effect of *S. filiforme* supplementation on the F/B ratio under either diet.

### 2.5. Taxonomic Structure of the Bacterial Communities

**Figure 8:**
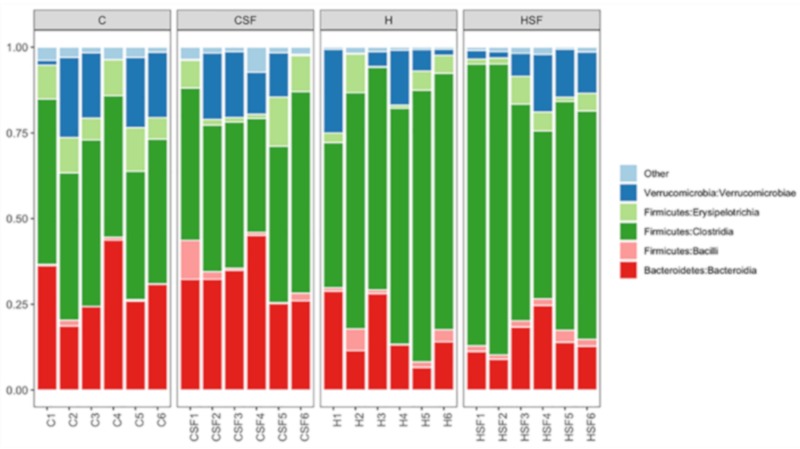
Taxonomic profiles of bacterial communities shown at the class level of all faecal samples. C, corn starch diet-fed rats; CSF, corn starch diet-fed rats supplemented with *Sarconema filiforme*; H, high-carbohydrate, high-fat diet-fed rats; and HSF, high-carbohydrate, high-fat diet-fed rats supplemented with *Sarconema filiforme*.

The most abundant bacterial classes found in the faecal samples for different treatment groups were Bacteroidia, Bacilli, Clostridia, Erysipelotrichia and Verrucomicrobia (Figure 8). Other bacterial classes, including Actinobacteria, Coriobacteriia, Melainabacteria, Deferribacteres, Saccharimonadia, Alphaproteobacteria, Deltaproteobacteria, Gammaproteobacteria and Mollicutes, were observed at lower abundance levels (<1%) in some (but not all) faecal samples.

The relative abundance of bacteria from the class Bacteroidia was reduced in H rats (15.03% to 17.07%) compared to C rats (29.98% to 32.68%) (*p* < 0.001). An increase in the relative abundance of bacteria from class Bacilli was observed in CSF, H and HSF rats (2.01% to 2.89%) compared to the C rats (0.58%; *p* > 0.05). A higher abundance of bacteria from the class Clostridia was observed for H rats (66.51% to 68.76%) compared to C rats (43.47% to 44.59%) (*p* < 0.0001) (Figure 8). An increase in the relative abundance of bacteria from the class Verrucomicrobiae and Erysipelotrichia was observed in C rats (Verrucomicrobiae: 10.74% to 13.92%; Erysipelotrichia: 6.17% to 9.30%) (*p* > 0.05) compared to H rats (Verrucomicrobiae: 8.83% to 9%; Erysipelotrichia: 3.86% to 4.33%) (*p* > 0.05; Figure 8).

**Figure 9:**
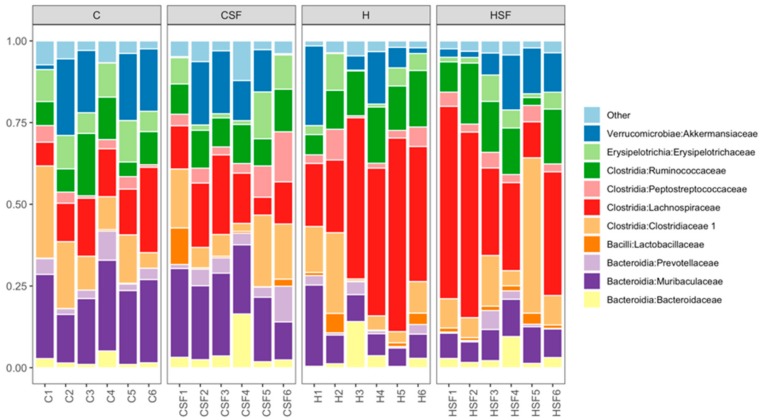
Taxonomic profiles of bacterial communities shown at the family level of all faecal samples. C, corn starch diet-fed rats; CSF, corn starch diet-fed rats supplemented with *Sarconema filiforme*; H, high-carbohydrate, high-fat diet-fed rats; HSF, high-carbohydrate, high-fat diet-fed rats supplemented with *Sarconema filiforme*.

Analysis of the bacterial community structure at the family level showed that Bacteroidaceae (class Bacteriodia), Muribaculaceae (class Bacteriodia), Prevotellaceae (class Bacteroidia), Lactobacillaceae (class Bacilli), Clostridiaceae 1 (class Clostridia), Lachnospiraceae (class Clostridia), Peptostreptococcaceae (class Clostridia), Ruminococcaceae (class Clostridia), Erysipelotrichaceae (class Erysipelotrichia) and Akkermansiacaeae (class Verrucomicrobia) were most dominant in the faecal samples (Figure 9). The relative abundance of bacteria from the family Ruminococcaceae was reduced for H rats (8.83% to 9%) compared to C rats (10.74% to 13.92%) (*p* > 0.05). A high abundance of bacteria from the family Lachnospiraceae was detected in H rats and HSF rats (36.37% to 39.43%, *p* < 0.0001) compared to C rats (15.17% to 15.26%). In contrast, the abundance of bacteria from the family Muribaculaceae was reduced in H rats (9.16% to 10.28%) compared to C rats (21.27% to 22.77%, *p* < 0.05) (Figure 9). Moreover, lower abundance of bacteria from the family Lactobacillaceae was observed for C rats (0.16%) compared to CSF, H and HSF (1.56% to 2.49%) (*p* > 0.05).

**Figure 10:**
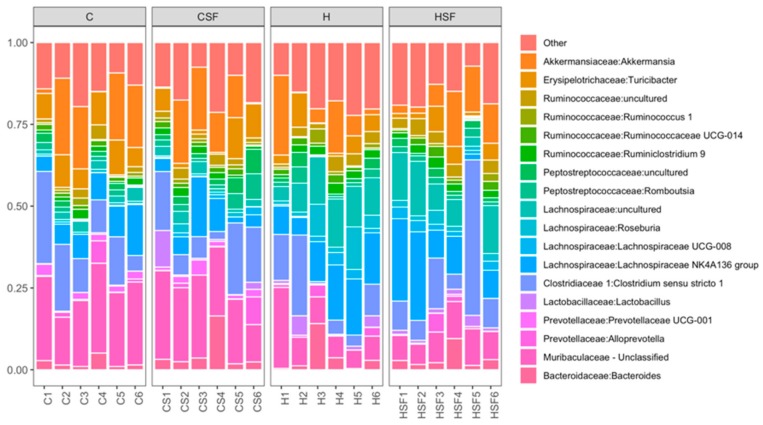
Taxonomic profiles of bacterial communities shown at the genus level of all faecal samples. C, corn starch diet-fed rats; CSF, corn starch diet-fed rats supplemented with *Sarconema filiforme*; H, high-carbohydrate, high-fat diet-fed rats; HSF, high-carbohydrate, high-fat diet-fed rats supplemented with *Sarconema filiforme*.

Analysis of the bacterial community structure at the genus level showed that *Bacteroides* (family Bacteroidaceae), unclassified Muribaculaeceae, *Clostridium sensu stricto 1* (family Clostridiaceae), *Lachnospiraceae* NK4A136 group (family Lachnospiraceae), *Roseburia* (family Lachnospiraceae), *Ruminococcus 1* (family Ruminococcaceae), unclassified Ruminococcaceae, *Turicibacter* (family Erysipelotrichaceae) and *Akkermansia* (family Akkermansiaceae) were most dominant in the faecal samples (Figure 10).

### 2.6. Multivariate Analysis of Physiological Data

A total of 23 physiological parameters were assessed and included in the analysis below (body weight, fat mass, lean mass, water intake, food intake, energy intake, feed efficiency, left ventricle with septum wet weight, right ventricle wet weight, retroperitoneal fat, omental fat, epididymal fat, total abdominal fat, liver wet weight, kidney wet weight, spleen wet weight, plasma non-esterified fatty acids, plasma triglycerides, systolic blood pressure, oral glucose tolerance area under the curve, oral glucose tolerance 120 min blood glucose concentrations, plasma aspartate transaminase activity and plasma alanine transaminase activity) for rats fed with the C and H diets and supplemented with *S. filiforme* (Table 4).

**Figure 11:**
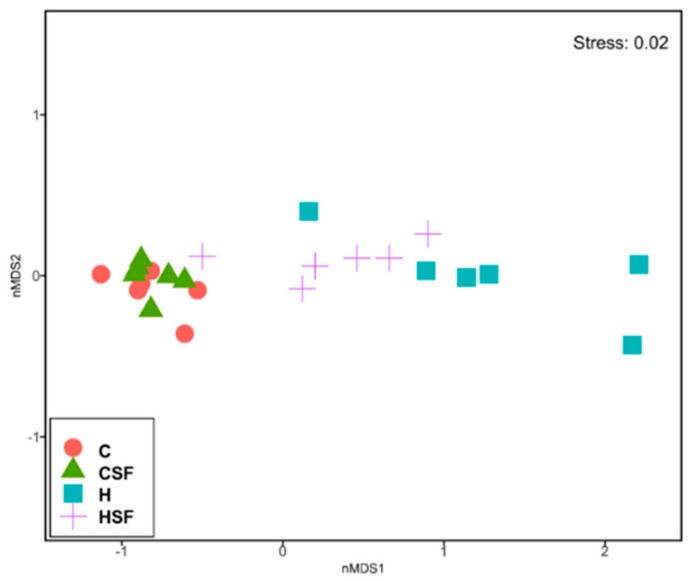
Non-metric multi-disciplinary scaling (nMDS) plot of physiological data from all physiological parameters measured after 16 weeks of feeding. C, corn starch diet-fed rats; CSF, corn starch diet-fed rats supplemented with *Sarconema filiforme*; H, high-carbohydrate, high-fat diet-fed rats; HSF, high-carbohydrate, high-fat diet-fed rats supplemented with *Sarconema filiforme*.

Distance-based multivariate analysis showed that treatments have distinct responses on the physiological parameters. Diet and supplement affected the rats’ physiological variables (Figure 11, Table 4; PERMANOVA; *p* = 0.0001 and *p* = 0.0154, respectively) and there was an interaction between the two factors (Figure 11, Table 4; PERMANOVA, *p* = 0.0187).

There was statistical support for differences between C and H rats without *S. filiforme* (*p* = 0.0018, Table 4) indicating an effect of basal diet on the overall physiological variables. There was also an effect for the addition of *S. filiforme* to the H diet (*p* = 0.0268; Figure 11, Table 4), however supplementation had no effect for C diet. Rat physiological variables in H and HSF rats were more variable between replicates compared to the CSF rats (Figure 11, Table 4; PERMDISP; *p* = 0.0398, *p* = 0.0139, respectively).

### 2.7. Differentially Abundant zOTUs under Different Feeding Treatments

Multivariate analysis of individual zOTUs using the R package Mvabund revealed that diet and supplement, as well as the interaction between diet and supplement had a significant effect on the bacterial community structure in the faecal samples (Table 5). At the zOTU level, diet had a stronger effect than supplementation with *S. filiforme* on the bacterial community structure by affecting the abundance of 77 zOTUs (6.24% of total zOTUs) (Table 5).

zOTUs belonging to the phylum Firmicutes (families: Lachnospiraceae, Peptococcaceae and Ruminococcaceae; genus: *Acetatifactor*, *Anaerostipes*, *Blautia*, *GCA-900066575*, *Lachnoclostridium*, *Lachnospiraceae* FCS020 group, *Lachnospiraceae* NK4A136 group, *Lachnospiraceae* UCG-006, *Lachnospiraceae* UCG-008, *Roseburia*, unclassified Lachnospiraceae, unclassified Peptococcaceae, *Butyricicoccus*, *Ruminiclostridium*, *Ruminiclostridium 9* and unclassified Ruminococcaceae) were reduced in abundance or absent in C and CSF rats compared to H and HSF rats, respectively, while zOTUs belonging to the family Ruminococcaceae (genus: *Ruminococcaceae* NK4A214 group) were enriched in C and CSF rats compared to H and HSF rats (Table 6). Bacteria belonging to the phylum Actinobacteria (families: Bifidobacteriaceae and Eggerthellaceae; genus: *Bifidobacterium* and *Enterorhabdus*) and the phylum Bacteroidetes (families: Bacteroidaceae, Muribaculaceae and Prevotellaceae; genus: *Bacteroides*, unclassified Muribaculaceae and *Prevotellaceae* UCG-001) were either reduced or absent in H and HSF rats (Table 6).

A total of four zOTUs (0.32% of total zOTUs) belonging mostly to phylum Firmicutes were significantly affected by *S. filiforme* supplementation (Table 7). One zOTU belonging to the phylum Bacteroidetes was enriched in C and H rats, while bacteria belonging to the phylum Firmicutes and family Ruminococcaceae (genus: *Ruminococcaceae* UCG-014) and the phylum Proteobacteria and family Desulfovibrionaceae (genus: *Bilophila*) were absent in C and H rats (Table 7).

### 2.8. Correlation of Microbiota and Physiological Parameters

**Figure 12:**
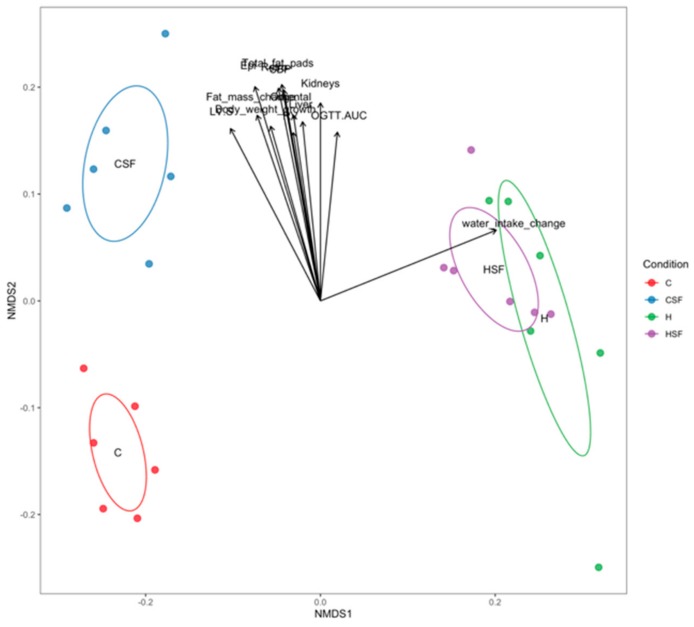
Correlation between bacterial community structure (points) and environmental variables (arrows). C, corn starch diet-fed rats; CSF, corn starch diet-fed rats supplemented with *Sarconema filiforme*; H, high-carbohydrate, high-fat diet-fed rats; HSF, high-carbohydrate, high-fat diet-fed rats supplemented with *Sarconema filiforme*.

Combined analysis of bacterial community structure and physiological parameters was performed. The Mantel test revealed that overall the bacterial community structure and the physiological data are correlated (Mantel statistic r = 0.2177; *p* = 0.0071). Table 8 and Figure 12 show how individual physiological parameters contribute to the differences in bacterial community structure between treatments (function envfit – vegan R package).

Physiological variables were further correlated with individual zOTUs (Table 9). A total of 44 zOTUs were found to be statistically correlated with at least one of the physiological parameters (*p* < 0.05). Of the zOTUs, 33 out of 44 belonged to the phylum Firmicutes, nine zOTUs to the phylum Bacteroidetes, one zOTU to the phylum Proteobacteria and one zOTU to the phylum Actinobacteria. Food intake (7 of 44 zOTUs or 15.91%) was inversely correlated with the relative abundance of the selected zOTU. In contrast, water intake (16 of 44 zOTUs or 36.36%), epididymal fat (4 of 44 zOTUs or 9.1%), left ventricle and septum weight (3 of 44 zOTUs or 6.82%), oral glucose tolerance (3 of 44 zOTUs or 6.82%), systolic blood pressure (3 of 44 zOTUs or 6.82%), liver wet weight (3 of 44 zOTUs or 6.82%) and total abdominal fat (2 of 44 zOTUs or 4.55%) were positively correlated with the selected zOTUs (Table 9).

The relative abundances of zOTUs belonging to the phylum Firmicutes and the families Lachnospiraceae (genus: *Anaerostipes*, *Blautia*, *Lachnospiraceae* FCS020 group, *Lachnospiraceae* NK4A136 group, *Roseburia*, unclassified Lachnospiraceae: zOTU77, zOTU244, zOTU856, zOTU762, zOTU37, zOTU556, zOTU582, 174, zOTU25, zOTU198, zOTU52 and zOTU590) and Ruminococcaceae (genus: *Rumiclostridium* and *Rumiclostridium 9*: zOTU279, zOTU614, zOTU133, zOTU135 and zOTU63) were inversely correlated with food intake and left ventricle and septum weight and positively correlated with water intake. While bacteria belonging to the phylum Firmicutes and family Peptococcaceae (zOTU398) were negatively correlated to water intake, bacteria belonging to the family Ruminococcaceae UCG-014 (zOTU595, zOTU232) were positively correlated to several physiological parameters including epididymal fat, retroperitoneal fat, right ventricle weight, systolic blood pressure and liver wet weight.

The relative abundances of bacteria belonging to the phylum Actinobacteria and family Bifidobacteriaceae; the phylum Bacteroidetes and families Bacteroidaceae, Muribaculaceae and Prevotellaceae (for example: zOTU42, zOTU1036, zOTU1144, zOTU21, zOTU27, zOTU79 and zOTU857) were negatively correlated to water intake. Several zOTUs belonging to the phylum Bacteroidetes and families Bacteroidaceae and Prevotellaceae were also positively correlated with left ventricle and septum weight (zOTU20 and zOTU10). The relative abundance of bacteria belonging to the phylum Proteobacteria and family Desulfovibrionaceae was positively correlated to epididymal fat, kidney weight, liver weight, oral glucose tolerance 120-minute blood glucose, oral glucose tolerance area under the curve, omental fat, retroperitoneal fat, systolic blood pressure and total abdominal fat (zOTU40).

## 3. Discussion

This project demonstrates that local Australian cultivation of *S. filiforme* produced significant and reliable yields of biomass in intensive tank-based culture, which can therefore potentially be a source of commercial quantities of ι-carrageenan. Australia has unique and untapped seaweed resources [11]; however, as of 2014, the Australian seaweed industry was small and only based on the harvest of stormcast kelp for alginate and fertiliser and on introduced species of *Undaria* for the extraction of bioactive compounds [12]. However, indigenous species such as *S. filiforme* could supply high-value fresh and dried foods as well as compounds for the nutraceutical and pharmaceutical markets. Consuming locally grown foods is considered important, because it reduces transport to markets and environmental impact to decrease CO_2_ emissions. In addition, local production supports the local economy as evidenced by the successful commercial cultivation of the red seaweed, *K. alvarezii*, in countries such as the Philippines and Indonesia as a main source of carrageenan for the food industry for more than 40 years [13].

Further, we show that whole dried *S. filiforme* may be useful in reversing metabolic syndrome. Metabolic syndrome including abdominal obesity, hypertension, hyperglycaemia, fatty liver and inflammation increases the risk of cardiovascular disease and diabetes; this syndrome is mimicked by a diet high in simple sugars, saturated and *trans* fats in rats [14]. This validated dietary model of human metabolic syndrome has been previously reported to show reversal of changes by interventions with seaweeds [15,16]. The major findings from the current reversal study were that *S. filiforme* decreased metabolic, cardiovascular and liver changes by 9-40% in obese rats fed a high-carbohydrate, high-fat diet, including some variables that were effectively reversed including liver enzymes and systolic blood pressure. These results are consistent with our previous study where *K. alvarezii* containing κ-carrageenan was used in a prevention protocol [8]. The soluble fibre content, which also approximates the carrageenan content, was ~35% in *K. alvarezii* and ~12% in *S. filiforme*, giving a soluble fibre content in the rat diets of around 1.7% and 0.5%, respectively. This was markedly less than the upper limit of 5% to avoid any safety risks, as recommended by the Scientific Committee on Food of the European Commission, for carrageenans below a molecular weight limit of 50 kDa when used as a food additive [17]. Although carrageenans have been used in subcutaneous injections to induce rat paw oedema as a model of inflammation [18], there have been dietary studies with no adverse effects using 5% ι-carrageenan prepared from *E. denticulatum* (previously *E. spinosum*) [19,20], although systemic administration of carrageenan increased biliary antibody titre, which is suggestive of bacterial intrusion [20,21]. In the literature, there are conflicting reports on the effects of carrageenans on the gastrointestinal tract, although the confusion may be in part due to inconsistent nomenclature. Degraded carrageenans have been incorrectly referred to as poligeenans, which are not produced biologically. Poligeenans are produced in the laboratory or commercially by subjecting carrageenan to very low pH at 0.9–1.3 and non-physiological temperatures of >80°C for several hours [22]. As carrageenans are not absorbed from the gastrointestinal tract after oral administration, studies using systemic administration are not appropriate for a risk assessment of carrageenans when used as food products in subjects without gastrointestinal pathology [23]. However, oral studies can determine local gastrointestinal risk; the current study shows no histopathological impact on the ileum or colon in H rats compared to HSF rats, consistent with the previous study on oral administration of *K. alvarezii* containing κ-carrageenan [8].

Traditionally, people in East Asian countries, such as Korea, Japan and China, consume more seaweeds as food and ingredients of traditional medicine than other populations [24]. The average intake of seaweeds in Japan was 14.3 g (wet weight)/day [25]. In the current study, the dose in rats equates to approximately 6.3 g of seaweed/day for humans [10]. Therefore, it is realistic that this quantity can be consumed as a single seaweed such as *S. filiforme* in the usual diet to translate its health benefits. Further, production of therapeutic amounts of this seaweed requires a small area, as the 42 m^2^ research facility of the University of the Sunshine Coast could provide enough seaweed for 179 people to be treated continuously, based on measured seaweed growth rates. This is markedly more efficient usage of space than with other sources of dietary fibre such as cereals; as an example, the average cereal yield in the USA was 828 g/m^2^/year in 2017 [26] compared to the average yield of seaweed at the University of the Sunshine Coast of around 189 g/m^2^/week (or around 10 kg/m^2^/year). Scaling up to produce commercial amounts of seaweed is therefore realistic.

Dietary fibre is classified as either soluble (non-cellulosic, polysaccharides, oligosaccharides, pectins, β-glucans and gums) or insoluble (cellulose, hemicellulose and lignin). The major physiological effects of soluble fibre are delayed gastric emptying, regulation of blood glucose levels and lowered serum cholesterol concentrations, due to increasing gut content viscosity and colonic fermentation. In contrast, the major effects of insoluble fibre are shortened gut transit time and improved laxation, both due to faecal bulking capacity and support for the growth of intestinal bacteria due to colonic fermentation [27]. *S. filiforme* contains about 22% fibre, which equates to 1.3 g of fibre per day if the human dose is 6.3 g/day, based on the Reagan-Shaw rat to human scaling equation [10]. The American Dietetic Association recommended a daily dietary fibre intake of 25 g for adult females and 38 g for adult males [28]. Therefore, *Sarconema* at this dose would increase fibre consumption, but alone would not provide sufficient fibre to meet this recommendation; consequently, other fibre-rich foods such as fruits, vegetables, beans and grains would be necessary components of the diet. Epidemiological and clinical studies have demonstrated that dietary fibre intake is inversely related to obesity [29], type 2 diabetes [30] and cardiovascular disease [31]. The consumption of dietary fibre likely reduces body fat accumulation due to several factors such as fermentation of the fibre in the colon, stimulating production of glucagon-like-peptide-1 and peptide YY [32]. These gut hormones increase satiety, which may lead to decreased meal frequency and size. Consumption of insoluble fibre reduced body weight and body fat and also normalised fasting glucose and insulin concentrations in overweight and obese adults [33].

Carrageenans undergo minimal digestion in the stomach and are then fermented by colonic bacteria [34], hence meeting the definition of prebiotics [35]. Health benefits of prebiotics include decreased blood pressure and body weight [27,36] similar to the responses from the current study. Using the same rat model of diet-induced obesity, a prebiotic mixture of inulin and oligofructose was reported as an effective dietary fibre, reducing body weight, plasma concentrations of free fatty acids and triglycerides, and systolic blood pressure and attenuating inflammatory cell infiltration in the heart and liver [37]. Red seaweeds show responses that suggest the biological actions of fibre as prebiotics. Subjects supplemented with the red seaweed *Chondrus crispus* showed improved gut health and immune modulation [38]. Another red seaweed, *Mastocarpus stellatus*, with 31.7% dietary fibre reduced triglycerides and total cholesterol concentrations, however, there was no effect on body weight in healthy Wistar rats [39]. Complex polysaccharides exert their action through a wide range of mechanisms including selective fermentation, lowering the gut pH, faecal bulking, preventing gut colonisation by pathogens, controlling putrefactive bacteria, and therefore reducing the host’s exposure to toxic metabolites [40]. These effects are likely due to dietary fibre increasing short-chain fatty acid production as these are used as an energy source by selected gut microbiota. Short-chain fatty acids decrease the luminal pH, improve calcium and magnesium absorption, reduce potential pathogenic bacteria and act as an energy source for epithelial cells [41].

## 4. Materials and Methods

### 4.1. *Sarconema filiforme* Source and Analysis

*S. filiforme* was cultured at the University of the Sunshine Coast seaweed aquaculture facility at the Bribie Island Research Centre, Woorim, QLD, Australia (27°04′10″ S; 153°12′15″ E) from January to March 2018. Production (yield of dry weight/m^2^/day) was measured through weekly harvests from up to five 1000 L fibreglass tanks over 13 weeks from cultures initially stocked each week at, on average, 7 g fresh weight per 1000 L of seawater. Cultures received flow-through seawater with weekly addition of nutrients (MAF, Manutech, Cavan, SA, Australia). Fresh samples were harvested each week, dehydrated and a total of 3 kg dried and milled *S. filiforme* was sub-sampled across the three months of production and stored in vacuum-sealed bags containing silica desiccant. Compositional analysis of the seaweed was performed as detailed previously [42,43].

Attenuated Total Reflectance-Fourier-Transform Infrared Spectroscopy (ATR-FTIR) was performed at Southern Cross University, Lismore, NSW, Australia to determine carrageenan subtypes in *S. filiforme* and *K. alvarezii* as a control and commercial grade ι-carrageenan and κ-carrageenan (Sigma-Aldrich Australia, Castle Hill, NSW, Australia) as standards. The transmittance spectra were recorded from 2000 to 675 cm^−1^ [44].

### 4.2. Rats and Diets

All experimental protocols were approved by the Animal Ethics Committee of the University of Southern Queensland under the guidelines of the National Health and Medical Research Council of Australia. Male Wistar rats (8–9 weeks old; 338 ± 1 g, n = 48) were obtained from the Animal Resource Centre, Murdoch, WA, Australia. Rats were individually housed in a temperature-controlled (21 ± 2˚C), 12-hour light/dark conditions with free access to food and water. Rats were randomly distributed into four groups, each of 12 rats. Two groups were fed either corn starch or high-carbohydrate, high-fat diets (C and H, respectively) [14] for a full 16 weeks. The other two groups received C and H diets for the first eight weeks and then received 5% *S. filiforme* in the diet for the last eight weeks (CSF and HSF, respectively). The C diet contained 570 g of cornstarch, 155 g of powdered rat food (Specialty Feeds, Glen Forest, WA, Australia), 25 g of Hubble, Mendel and Wakeman salt mixture (MP Biomedicals, Seven Hills, NSW, Australia), and 250 g of water per kilogram of diet. The H diet contained 175 g of fructose, 395 g of sweetened condensed milk, 200 g of beef tallow, 155 g of powdered rat food (all obtained from local food suppliers and supermarkets), 25 g of Hubble, Mendel and Wakeman salt mixture and 50 g of water per kilogram of diet. In addition, the drinking water for the H and HSF groups was supplemented with 25% fructose [14].

### 4.3. Rat Measurements

Dual-energy X-ray absorptiometry, non-invasive systolic blood pressure, abdominal circumference, oral glucose and insulin tolerance tests and indirect calorimetry were performed as described [14]. Euthanasia followed by heparin injection, blood collection, centrifugation, storage and then isolated Langendorff heart preparation and measurements, plasma measurements, organ weights, organ bath study and histological analyses were performed as described [14].

### 4.4. Gut Microbiota Analysis

Immediately after euthanasia and organ removal, two or three faecal pellets were collected from the colon of rats and stored at −80 °C in nuclease-free tubes. Total microbial community DNA was extracted from faecal samples using the DNeasy Powersoil Kit (Qiagen Australia, Chadstone, VIC, Australia) following the manufacturer’s instructions [45]. The bacterial gut microbiota was then characterised by amplifying and sequencing the 16S rRNA gene. The primers, 341F (TCGTCGGCAGCGTCAGATGTGTATAAGAGACAGCCTACGGGNGGCWGCAG) and 785R (GTCTCGTGGGCTCGGAGATGTGTATAAGAGACAGGACTACHVGGGTATCTAATCC) were used to amplify the V3-V4 regions of the 16S rRNA gene, which was then sequenced on an Illumina MiSeq platform. Sequencing reads were processed to form zOTUs, which were taxonomically classified against the SILVA database.

The reaction mixture (50 μL total volume per sample) to amplify the 16S rRNA gene consisted of Econotaq® PLUS GREEN 2× Master Mix (Astral Scientific, Gymea, NSW, Australia) (25 uL), Ambion® nuclease-free water (17 μL), the primer pair 341F and 785R (1.5 μL of each; 10 μM) and DNA template (5 μL). The PCR program consisted of an initial denaturation at 94 °C (2 min), followed by 35 cycles of denaturation at 94^o^C (30 s), annealing at 55^o^C (30 s), extension at 72 °C (40 s) and a final extension at 72 °C (7 min). PCR products were then quantified using gel electrophoresis. Paired-end sequencing (2 × 300 bp) of the resulting 16S rRNA gene amplicons was performed at the Ramaciotti Centre for Genomics, University of New South Wales on an Illumina MiSeq platform following the MiSeq System User Guide [46]. For 16S rRNA gene sequencing analysis, sequence data were initially quality-filtered and trimmed using Trimmomatic version 0.36 truncating reads if the quality dropped below 20 in a sliding window of 4 bp [47]. USEARCH version 11.0.667 [48] was used for further processing [49] to merge and quality-filter sequencing reads, excluding reads with < 250 or > 550 nucleotides, in addition to reads with more than one ambiguous base or an expected error of more than 1. Filtered sequences were denoised and clustered into unique sequences (zero-radius operational taxonomic units; zOTUs) using the UNOISE algorithm [50] implemented in USEARCH. zOTUs represent unique bacterial entities and roughly are equivalent to species or strains. Chimeric sequences were removed *de novo* during clustering and subsequently in reference mode using UCHIME [51] with the SILVA database (https://www.arb-silva.de/browser/) (SILVA SSURef 132 NR) as a reference [52]. zOTUs were then taxonomically classified (i.e., assigned a likely taxonomic name) by BLASTN [53] against the SILVA database. All non-bacterial zOTUs were removed along with non-BLAST aligned and singleton zOTUs. Finally, processed sequences were mapped on zOTU sequences to calculate the distribution and counts of each zOTU in every sample. Only zOTUs occurring in more than two samples were considered for further statistical analysis.

### 4.5. Statistical Analysis

Physiological and metabolic data are presented as mean ± standard error of the mean (SEM). These results were tested for variance using Bartlett’s test and variables that were not normally distributed were transformed using log10 function prior to statistical analyses. Data from the four groups were tested by two-way analysis of variance. When the interactions and/or the main effects were significant, means were compared using the Newman-Keuls multiple comparison *post hoc* test. Where transformations did not result in normality or constant variance, a Kruskal-Wallis non-parametric test was performed. A *p* value of < 0.05 was considered as statistically significant. All statistical analyses were performed using Prism version 5.00 for Windows (GraphPad Software, San Diego, CA, USA).

For microbiota results, rarefaction curves were generated using the *rarecurve* function in *vegan* [54] and used to determine if a complete representation of the sample’s microbiota had been achieved given the sequencing effort. Prior to further analysis, the numbers of sequences were standardised across samples to account for different sequencing depths by randomly subsampling each sample to the lowest number of sequences counts obtained for any given sample (i.e., 19,706 counts). Bacterial alpha-diversities (zOTU richness and Shannon’s diversity) were calculated in R (version 3.5.3) using the rrarefy function in the vegan package [55]. A one-way analysis of variance test in GraphPad Prism 8.0.2 (San Diego, CA, USA) followed by Tukey’s pairwise comparisons test was used to determine the significance between the different groups and a *p* value of <0.05 was considered to be significant.

For multivariate analysis of bacterial communities, zOTU tables were imported into PRIMER [55] to compare the community structure (i.e. relative abundance data). Bray-Curtis similarity coefficients were calculated using square-root transformed zOTU abundances and the resulting similarity matrix was visualised using non-metric, multi-dimensional scaling (nMDS). Permutational multivariate analysis of variance (PERMANOVA) [56] with 9,999 random permutations was used to test the effect of treatment on bacterial communities in rat faecal samples.

## 5. Conclusions

Rats fed a high-carbohydrate, high-fat diet supplemented with *S. filiforme* as a source of *ι*-carrageenan decreased body weight, systolic blood pressure, abdominal and liver fat and plasma total cholesterol concentrations compared to H controls. These results are comparable to our previous study with *K. alvarezii* as a source of *κ*-carrageenan, providing evidence that red seaweeds contain compounds such as sulfated polysaccharides (carrageenans) which attenuate symptoms of diet-induced metabolic syndrome in rats. The correlations between changes in the gut microbiota and physiological changes following administration of *S. filiforme* suggests that the mechanism is likely through carrageenans acting as prebiotics as well as systemic anti-inflammatory responses in organs such as heart and liver. Further studies with mechanistic analyses will be valuable to determine the actions of carrageenans *in vivo* that are responsible for their health benefits.

## Figures and Tables

**Table 1:** Biochemical composition of *Sarconema filiforme* biomass.

**Proximate**	**% dry weight**	**Metals**	**mg/kg**
Carbohydrate	34.4		
Protein	11.8	Aluminium	39.3
Lipid	1.4	Antimony	0.1
Ash	50.8	Arsenic	6.2
Moisture	1.6	Barium	0.5
Energy (kJ/g)	8.7	Boron	299
**Fibre**	**% dry weight**	Cadmium	0.1
Total dietary fibre	21.7	Calcium	2150
Insoluble dietary fibre	9.6	Chromium	0.3
Soluble dietary fibre (by difference)	12.2	Cobalt	0.6
**Ultimate**	**% dry weight**	Copper	1.6
Carbon	21.2	Iron	1875
Nitrogen	2.6	Lead	0.2
Hydrogen	3.3	Magnesium	3655
Sulfur	4.2	Manganese	20.3
**Amino acids**	**% dry weight**	Mercury	0.3
Total amino acids	11.8	Molybdenum	0.2
Histidine	0.2	Nickel	0.7
Serine	0.7	Phosphorus	2500
Arginine	1.0	Potassium	189,500
Glycine	0.7	Selenium	0.3
Aspartic acid	1.3	Silver	0.0
Glutamic acid	1.8	Sodium	27,000
Threonine	0.6	Strontium	31.3
Alanine	0.8	Tin	0.1
Proline	0.6	Vanadium	2.4
Lysine	0.6	Zinc	128
Tyrosine	0.3	**Fatty acids**	**% dry weight**
Methionine	0.2		
Valine	0.8	Total fatty acids	1.1
Isoleucine	0.7	C16:0 palmitic	0.4
Leucine	1.0	C20:4 ω-6 arachidonic	0.5
Phenylalanine	0.6	C20:5 ω-3 eicosapentaenoic	0.1

Values are mean of two sub-samples of blended biomass.

**Table 2:** Responses to *Sarconema filiforme*.

Variables	C	CSF	H	HSF	*p* Value		
					Diet	Treatment	Interaction
**Physiological variables**							
0 week body weight, *g*	338 ± 1	338 ± 1	339 ± 1	338 ± 1	0.66	0.66	0.66
8 week body weight, *g*	371 ± 11^b^	359 ± 5^b^	451 ± 15^a^	442 ± 10^a^	<0.0001	0.31	0.88
16 week body weight, *g*	405 ± 4^c^	398 ± 5^c^	550 ± 15^a^	498 ± 10^b^	<0.0001	0.004	0.025
16 week lean mass, *g*	321 ± 9	324 ± 7	309 ± 10	338 ± 12	0.92	0.13	0.22
16 week fat mass, *g*	59 ± 4^c^	58 ± 6^c^	251 ± 30^a^	151 ± 16^b^	<0.0001	0.004	0.005
8 week lean/fat mass proportion	4.4 ± 0.5^b^	6.8 ± 0.8^a^	2.2 ± 0.4^c^	2.3 ±0.2^c^	<0.0001	0.056	0.08
16 week lean/fat mass proportion	5.5 ± 0.4^b^	6.0 ± 0.6^a^	1.3 ± 0.1^d^	2.5 ± 0.4^c^	<0.0001	0.047	0.93
16 week bone mineral content, *g*	12.3 ± 0.8^c^	12.1 ± 0.5^c^	17.6 ± 0.7^a^	14.6 ± 0.6^b^	<0.0001	0.021	0.041
16 week bone mineral density, *g/cm^2^*	0.180 ± 0.002	0.183 ± 0.004	0.184 ± 0.005	0.184 ± 0.002	0.53	0.70	0.70
Food intake 0-8 weeks, *g/day*	41.4 ± 0.9^a^	39.0 ± 1.4^a^	27.8 ± 0.9^b^	26.5 ± 1.3^b^	<0.0001	0.21	0.71
Food intake 9-16 weeks, *g/day*	37.1 ± 0.4^a^	37.0 ± 1.1^a^	26.2 ± 0.7^b^	20.9 ± 0.8^c^	<0.0001	0.012	0.015
Water intake 0-8 weeks, *g/day*	40.8 ± 4.4^a^	32.7 ± 2.8^b^	25.5 ± 0.8^c^	29.3 ± 2.7^b^	0.0061	0.51	0.07
Water intake 9-16 weeks, *g/day*	30.4 ± 3.8^b^	42.3 ± 1.4^a^	22.2 ± 0.6^c^	41.8 ± 2.1^a^	0.07	<0.0001	0.11
Energy intake 0-8 weeks, *kJ/day*	468 ± 9^b^	438 ±16^b^	593 ± 13^a^	597 ± 35^a^	<0.0001	0.65	0.55
Energy intake 9-16 weeks, *kJ/day*	415 ± 4^b^	407 ± 13^b^	558 ± 10^a^	533 ± 17^a^	<0.0001	0.18	0.93
16 week abdominal circumference, *cm*	20.1 ± 0.4^c^	19.4 ± 0.1^c^	23.8 ± 0.5^a^	21.2 ± 0.3^b^	<0.0001	<0.0001	0.005
Body mass index, *g/cm^2^*	0.61 ± 0.02^c^	0.65 ± 0.01^c^	0.81 ± 0.03^a^	0.74 ± 0.02^b^	<0.0001	0.46	0.011
Retroperitoneal fat, *mg/mm*	216 ± 37^c^	196 ± 15^c^	636 ± 67^a^	423 ± 41^b^	<0.0001	0.007	0.025
Epididymal fat, *mg/mm*	78 ± 18^c^	62 ± 9^c^	191 ± 28^a^	116 ± 12^b^	<0.0001	0.007	0.07
Omental fat, *mg/mm*	147 ± 21^c^	142 ± 12^c^	333 ± 29^a^	245 ± 16^b^	<0.0001	0.019	0.035
Visceral adiposity, *%*	5.1 ± 0.6^c^	4.8 ± 0.4^c^	10.0 ± 0.6^a^	7.8 ± 0.3^b^	<0.0001	0.011	0.048
Liver wet weight, *mg/mm*	218 ± 7^b^	231 ± 7^b^	365 ± 26^a^	343 ± 16^a^	<0.0001	0.77	0.27
***Cardiovascular variables***							
8 week systolic blood pressure, *mmHg*	123 ± 4^b^	122 ± 3^b^	135 ± 3^a^	138 ± 2^a^	0.0001	0.76	0.54
16 week systolic blood pressure, *mmHg*	128 ± 3^b^	126 ± 4^b^	145 ± 4^a^	132 ± 3^b^	0.007	0.07	0.18
Left ventricle + septum wet weight, *mg/mm*	22.6 ± 2.1	22.5 ± 1.0	21.2 ± 2.2	21.5 ± 0.6	0.38	0.94	0.88
Right ventricle, *mg/mm*	4.1 ± 0.3	3.5 ± 0.3	4.4 ± 0.1	4.3 ± 0.2	0.06	0.22	0.38
Left ventricular diastolic stiffness (κ)	21.2 ± 2.3^c^	21.7 ± 1.7^c^	29.6 ± 1.4^a^	25.6 ± 1.9^b^	0.022	0.36	0.24
Left ventricle collagen area, *%*	8.1 ± 2.4^c^	8.3 ± 1.8^c^	18.4 ± 1.9^a^	12.1 ± 1.7^b^	0.009	0.11	0.11
Left ventricle inflammatory cells, *cells/200µm^2^*	7 ± 2^c^	9 ± 2^c^	25 ± 3^a^	16 ± 3^b^	0.0004	0.1949	0.0520
***Metabolic variables***							
Plasma total cholesterol, *mmol/L*	1.59 ± 0.06^b^	1.44 ± 0.06^b^	1.73 ± 0.09^a^	1.51 ± 0.08^b^	0.18	0.021	0.65
Plasma triglycerides, *mmol/L*	0.50 ± 0.05^b^	0.52 ± 0.08^b^	1.15 ± 0.13^a^	1.04 ± 0.21^a^	<0.0001	0.73	0.62
Plasma non-esterified fatty acids, *mmol/L*	0.68 ± 0.12^b^	0.74 ± 0.24^b^	2.71 ± 0.29^a^	1.99 ± 0.59^a^	<0.0001	0.33	0.25
Alanine transaminase, *U/L*	39 ± 5^ab^	33 ± 5^ab^	50 ± 11^ab^	31 ± 2^b^	0.55	0.010	0.39
Aspartate transaminase, *U/L*	138 ± 20^ab^	125 ± 20^b^	174 ± 17^a^	128 ± 13^b^	0.29	0.011	0.37
Liver inflammatory cells, *cells/200µm^2^*	11 ± 2	13 ± 2	30 ± 3^a^	16 ± 3^b^	0.0003	0.029	0.005
Liver fat vacuoles area, *µm^2^*	12.4 ± 1.2	10.7 ± 1.8	90.6 ± 7.1^a^	70.4 ± 2.7^b^	<0.0001	0.012	0.029
**Oral glucose tolerance test**							
0 week basal blood glucose, *mmol/L*	2.6 ± 0.1	2.6 ± 0.1	2.6 ± 0.1	2.5 ± 0.1	0.66	0.66	0.66
0 week area under the curve, *mmol/L×min*	751 ± 26	650 ± 23	727 ± 23	689 ± 18	0.76	0.007	0.20
8 week basal blood glucose, *mmol/L*	2.5 ± 0.1^b^	2.8 ± 0.1^b^	3.9 ± 0.4^a^	3.4 ± 0.1^a^	<0.0001	0.60	0.044
8 week 120-min blood glucose, *mmol/L*	3.6 ± 0.4^b^	3.6 ± 0.1^b^	5.2 ± 0.1^a^	5.1 ± 0.1^a^	<0.0001	0.78	0.78
8 week area under the curve, *mmol/L×min*	559 ± 34^b^	538 ± 18^b^	690 ± 17^a^	645 ± 12^a^	<0.0001	0.11	0.56
16 week basal blood glucose, *mmol/L*	2.6 ± 0.1^b^	2.6 ± 0.2^b^	3.5 ± 0.3^a^	3.3 ± 0.2^a^	0.002	0.67	0.67
16 week 120-min blood glucose, *mmol/L*	3.6 ± 0.1^b^	3.8 ± 0.2^b^	5.8 ± 0.5^a^	5.0 ± 0.1^a^	<0.0001	0.22	0.043
16 week area under the curve, *mmol/L×min*	506 ± 22^c^	547 ± 8^b^	675 ± 20^a^	647 ± 18^a^	<0.0001	0.71	0.056
**Insulin tolerance test**							
8 week 120-min blood glucose, *mmol/L*	2.1 ± 0.7^b^	2.5 ± 0.5^b^	3.8 ± 0.5^a^	4.2 ± 0.4^a^	0.004	0.47	1.00
8 week area under the curve, *mmol/L×min*	204 ± 33^b^	238 ± 36^b^	349 ± 23^a^	377 ± 24^a^	0.0002	0.37	0.93
16 week 120-min blood glucose, *mmol/L*	3.6 ± 0.4	3.3 ± 1.1	4.3 ± 1.1	3.4 ± 0.3	0.66	0.52	0.74
16 week area under the curve, *mmol/L×min*	271 ± 64	225 ± 36	396 ± 56	341 ± 19	0.006	0.23	0.91

Values are presented as mean ± SEM, n = 10-12. Means in a row with unlike superscripts differ (a, b or c), *p* < 0.05. C, corn starch diet-fed rats; CSF, corn starch diet-fed rats supplemented with *Sarconema filiforme*; H, high-carbohydrate, high-fat diet-fed rats; HSF, high-carbohydrate, high-fat diet-fed rats supplemented with *Sarconema filiforme*.

**Table 3:** PERMANOVAs based on Bray-Curtis similarity measure for square-root transformed abundances of all rat faecal samples.

**PERMANOVA**						
**Source**	**df**	**SS**	**MS**	**Pseudo-F**	**P(perm)**	**Unique perms**
Diet	1	9306.2	9306.2	9.3671	0.0001	9912
Treatment	1	2477.6	2477.6	2.4938	0.0001	9860
Diet × treatment	1	1671.4	1671.4	1.6823	0.003	9836
Res	19	18876	993.5			
Total	22	32369				
**PAIR-WISE TESTS**						
**Source**				**t**	**P(perm)**	**Unique perms**
C, CSF				1.626	0.0014	462
C, H				2.4472	0.0017	462
C, HSF				2.544	0.0024	462
CSF, H				2.3327	0.0018	462
CSF, HSF				2.2125	0.0022	461
H, HSF				1.185	0.0396	462
**PERMDISP (PAIRWISE COMPARISONS)**						
**Groups**					**t**	**P(perm)**
C, CSF					1.7793	0.022
C, H					0.77115	0.65
C, HSF					0.80101	0.60
CSF, H					0.01072	0.99
CSF, HSF					1.6113	0.24
H, HSF					1.139	0.35

*p* values were calculated using 9,999 permutations under a residual model. C, corn starch diet-fed rats; CSF, corn starch diet-fed rats supplemented with *Sarconema filiforme*; H, high-carbohydrate, high-fat diet-fed rats; HSF, high-carbohydrate, high-fat diet-fed rats supplemented with *Sarconema filiforme*.

**Table 4:** PERMANOVAs based on Euclidean distance matrix for physiological data of all rat faecal samples.

**PERMANOVA**						
**Source**	**df**	**SS**	**MS**	**Pseudo-F**	**P(perm)**	**Unique perms**
Diet	1	4081700	4081700	53.366	0.0001	9924
Treatment	1	435050	435050	5.6882	0.0154	9942
Diet × treatment	1	408770	408770	5.3446	0.0187	9939
Res	20	1529700	76484			
Total	23	6455200				
**PAIR-WISE TESTS**						
**Source**				**t**	**P(perm)**	**Unique perms**
C, CSF				0.6848	0.82	462
C, H				5.6852	0.0018	461
C, HSF				4.4192	0.0024	462
CSF, H				5.8892	0.0021	462
CSF, HSF				4.7191	0.0023	462
H, HSF				2.5161	0.0268	462
**PERMDISP (PAIRWISE COMPARISONS)**						
**Groups**					**t**	**P(perm)**
C, CSF					1.5351	0.23
C, H					1.9906	0.11
C, HSF					1.4159	0.20
CSF, H					2.4448	0.0398
CSF, HSF					2.2429	0.0139
H, HSF					1.0507	0.29

*p* values were calculated using 9999 permutations under a residual model. C, corn starch diet-fed rats; CSF, corn starch diet-fed rats supplemented with *Sarconema filiforme*; H, high-carbohydrate, high-fat diet-fed rats; HSF, high-carbohydrate, high-fat diet-fed rats supplemented with *Sarconema filiforme*.

**Table 5:** Summary of statistical tests on differential zOTU abundance.

**Global Test (GLMs) by Mvabund**		
Diet: *p* < 0.0001		
Treatment: *p* = 0.003		
Diet × Treatment: *p* < 0.007		
**Univariate Analysis by Mvabund (*p* < 0.05)**		
***Factor***	***Number of differentially abundant OTUs***	***% of total number of OTUs***
Diet	77	6.24%
Treatment	35	0.32%
Total (unique zOTUs affected by one or more factors)	81	6.57%

**Table 6:** Relative abundance of zOTUs affected by diet (ANOVA with *p* adjusted <0.05) between C, CSF, H and HSF rats.

OTU_ID	C (%)	CSF (%)	H (%)	HSF (%)	Phylum	Family	Genus
Zotu42	0.62	0.31	0.00	0.02	Actinobacteria	Bifidobacteriaceae	*Bifidobacterium*
Zotu80	0.29	0.22	0.00	0.00	Actinobacteria	Bifidobacteriaceae	*Bifidobacterium*
Zotu168	0.12	0.09	0.01	0.01	Actinobacteria	Eggerthellaceae	*Enterorhabdus*
Zotu20	0.95	0.90	0.02	0.08	Bacteroidetes	Bacteroidaceae	*Bacteroides*
Zotu21	1.11	0.73	0.16	0.09	Bacteroidetes	Muribaculaceae	unclassified
Zotu27	1.05	0.37	0.05	0.06	Bacteroidetes	Muribaculaceae	unclassified
Zotu79	0.40	0.13	0.02	0.01	Bacteroidetes	Muribaculaceae	unclassified
Zotu541	0.02	0.05	0.00	0.00	Bacteroidetes	Muribaculaceae	unclassified
Zotu857	0.08	0.04	0.00	0.00	Bacteroidetes	Muribaculaceae	unclassified
Zotu978	0.12	0.11	0.01	0.03	Bacteroidetes	Muribaculaceae	unclassified
Zotu1036	0.05	0.02	0.00	0.00	Bacteroidetes	Muribaculaceae	unclassified
Zotu1144	0.12	0.04	0.00	0.01	Bacteroidetes	Muribaculaceae	unclassified
Zotu10	2.06	2.28	0.28	0.19	Bacteroidetes	Prevotellaceae	*Prevotellaceae* UCG-001
Zotu916	0.00	0.00	0.02	0.02	Firmicutes	Lachnospiraceae	*Acetatifactor*
Zotu77	0.04	0.05	0.30	0.54	Firmicutes	Lachnospiraceae	Anaerostipes
Zotu244	0.01	0.00	0.07	0.11	Firmicutes	Lachnospiraceae	*Blautia*
Zotu49	0.01	0.02	0.81	0.43	Firmicutes	Lachnospiraceae	GCA-900066575
Zotu76	0.03	0.03	0.64	0.20	Firmicutes	Lachnospiraceae	*Lachnoclostridium*
Zotu856	0.00	0.00	0.03	0.02	Firmicutes	Lachnospiraceae	*Lachnospiraceae* FCS020 group
Zotu1061	0.00	0.00	0.03	0.03	Firmicutes	Lachnospiraceae	*Lachnospiraceae* FCS020 group
Zotu37	0.01	0.01	0.81	1.19	Firmicutes	Lachnospiraceae	*Lachnospiraceae* NK4A136 group
Zotu100	0.36	0.14	0.00	0.02	Firmicutes	Lachnospiraceae	*Lachnospiraceae* NK4A136 group
Zotu123	0.00	0.00	0.20	0.40	Firmicutes	Lachnospiraceae	*Lachnospiraceae* NK4A136 group
Zotu182	0.00	0.00	0.18	0.23	Firmicutes	Lachnospiraceae	*Lachnospiraceae* NK4A136 group
Zotu201	0.01	0.01	0.18	0.13	Firmicutes	Lachnospiraceae	*Lachnospiraceae* NK4A136 group
Zotu544	0.00	0.00	0.13	0.09	Firmicutes	Lachnospiraceae	*Lachnospiraceae* NK4A136 group
Zotu561	0.00	0.00	0.06	0.03	Firmicutes	Lachnospiraceae	*Lachnospiraceae* NK4A136 group
Zotu658	0.00	0.00	0.48	0.54	Firmicutes	Lachnospiraceae	*Lachnospiraceae* NK4A136 group
Zotu762	0.00	0.00	0.02	0.04	Firmicutes	Lachnospiraceae	*Lachnospiraceae* NK4A136 group
Zotu847	0.00	0.00	0.03	0.02	Firmicutes	Lachnospiraceae	*Lachnospiraceae* NK4A136 group
Zotu966	0.00	0.00	0.08	0.10	Firmicutes	Lachnospiraceae	*Lachnospiraceae* NK4A136 group
Zotu1157	0.00	0.01	0.12	0.35	Firmicutes	Lachnospiraceae	*Lachnospiraceae* NK4A136 group
Zotu26	0.00	0.00	1.65	1.27	Firmicutes	Lachnospiraceae	*Lachnospiraceae* UGC-006
Zotu110	0.01	0.02	0.49	0.25	Firmicutes	Lachnospiraceae	*Lachnospiraceae* UGC-008
Zotu35	0.01	0.01	1.69	0.32	Firmicutes	Lachnospiraceae	*Roseburia*
Zotu556	0.00	0.00	0.02	0.04	Firmicutes	Lachnospiraceae	*Roseburia*
Zotu582	0.00	0.00	0.02	0.05	Firmicutes	Lachnospiraceae	*Roseburia*
Zotu604	0.00	0.00	0.01	0.05	Firmicutes	Lachnospiraceae	*Roseburia*
Zotu625	0.00	0.00	0.01	0.03	Firmicutes	Lachnospiraceae	*Roseburia*
Zotu634	0.00	0.00	0.01	0.05	Firmicutes	Lachnospiraceae	*Roseburia*
Zotu25	0.00	0.00	1.10	0.83	Firmicutes	Lachnospiraceae	unclassified
Zotu52	0.09	0.04	0.48	0.48	Firmicutes	Lachnospiraceae	unclassified
Zotu73	0.02	0.00	0.24	0.44	Firmicutes	Lachnospiraceae	unclassified
Zotu101	0.00	0.00	0.28	0.24	Firmicutes	Lachnospiraceae	unclassified
Zotu174	0.00	0.00	0.13	0.32	Firmicutes	Lachnospiraceae	unclassified
Zotu197	0.00	0.00	0.46	0.05	Firmicutes	Lachnospiraceae	unclassified
Zotu198	0.02	0.02	0.27	0.32	Firmicutes	Lachnospiraceae	unclassified
Zotu221	0.00	0.00	0.14	0.09	Firmicutes	Lachnospiraceae	unclassified
Zotu226	0.00	0.00	0.15	0.22	Firmicutes	Lachnospiraceae	unclassified
Zotu277	0.00	0.00	0.20	0.04	Firmicutes	Lachnospiraceae	unclassified
Zotu293	0.00	0.00	0.04	0.18	Firmicutes	Lachnospiraceae	unclassified
Zotu516	0.00	0.00	0.07	0.06	Firmicutes	Lachnospiraceae	unclassified
Zotu528	0.00	0.00	0.05	0.03	Firmicutes	Lachnospiraceae	unclassified
Zotu530	0.00	0.00	0.06	0.05	Firmicutes	Lachnospiraceae	unclassified
Zotu590	0.00	0.00	0.05	0.04	Firmicutes	Lachnospiraceae	unclassified
Zotu757	0.00	0.00	0.01	0.04	Firmicutes	Lachnospiraceae	unclassified
Zotu891	0.00	0.00	0.02	0.02	Firmicutes	Lachnospiraceae	unclassified
Zotu937	0.00	0.00	0.07	0.26	Firmicutes	Lachnospiraceae	unclassified
Zotu988	0.00	0.00	0.01	0.01	Firmicutes	Lachnospiraceae	unclassified
Zotu1043	0.01	0.02	0.23	0.17	Firmicutes	Lachnospiraceae	unclassified
Zotu1161	0.00	0.00	0.09	0.04	Firmicutes	Lachnospiraceae	unclassified
Zotu1200	0.00	0.01	0.33	0.16	Firmicutes	Lachnospiraceae	unclassified
Zotu247	0.00	0.00	0.13	0.04	Firmicutes	Peptococcaceae	unclassified
Zotu398	0.05	0.04	0.00	0.00	Firmicutes	Peptococcaceae	unclassified
Zotu384	0.00	0.00	0.10	0.02	Firmicutes	Ruminococcaceae	*Butyricicoccus*
Zotu279	0.00	0.00	0.07	0.08	Firmicutes	Ruminococcaceae	*Ruminiclostridium*
Zotu614	0.00	0.00	0.02	0.04	Firmicutes	Ruminococcaceae	*Ruminiclostridium*
Zotu958	0.00	0.00	0.01	0.01	Firmicutes	Ruminococcaceae	*Ruminiclostridium*
Zotu63	0.00	0.00	0.51	0.30	Firmicutes	Ruminococcaceae	*Ruminiclostridium 9*
Zotu133	0.01	0.00	0.15	0.23	Firmicutes	Ruminococcaceae	*Ruminiclostridium 9*
Zotu135	0.00	0.00	0.17	0.21	Firmicutes	Ruminococcaceae	*Ruminiclostridium 9*
Zotu643	0.01	0.04	0.00	0.00	Firmicutes	Ruminococcaceae	*Ruminiclostridium 9*
Zotu38	0.93	0.21	0.00	0.00	Firmicutes	Ruminococcaceae	*Ruminiclostridium* NK4A214 group
Zotu218	0.08	0.07	0.01	0.01	Firmicutes	Ruminococcaceae	*Ruminiclostridium* NK4A214 group
Zotu50	0.01	0.04	0.50	0.38	Firmicutes	Ruminococcaceae	unclassified
Zotu62	0.02	0.00	0.46	0.43	Firmicutes	Ruminococcaceae	unclassified
Zotu275	0.00	0.00	0.06	0.12	Firmicutes	Ruminococcaceae	unclassified

Differential abundance analysis was performed using Mvabund. C, corn starch diet-fed rats; CSF, corn starch diet-fed rats supplemented with *Sarconema filiforme*; H, high-carbohydrate, high-fat diet-fed rats; HSF, high-carbohydrate, high-fat diet-fed rats supplemented with *Sarconema filiforme*.

**Table 7:** Relative abundance of zOTUs affected by treatment (ANOVA with *p* adjusted <0.05) between C, CSF, H and HSF rats.

OTU_ID	C (%)	CSF (%)	H (%)	HSF (%)	Phylum	Family	Genus
Zotu15	0.72	0.00	3.02	0.00	Bacteroidetes	Muribaculaceae	unclassified
Zotu232	0.00	0.06	0.00	0.13	Firmicutes	Ruminococcaceae	Ruminococcaceae UCG-014
Zotu595	0.00	0.04	0.00	0.01	Firmicutes	Ruminococcaceae	Ruminococcaceae UCG-014
Zotu40	0.00	0.70	0.00	0.43	Proteobacteria	Desulfovibrionaceae	Bilophila

Differential abundance analysis was performed using Mvabund. C, corn starch diet-fed rats; CSF, corn starch diet-fed rats supplemented with *Sarconema filiforme*; H, high-carbohydrate, high-fat diet-fed rats; HSF, high-carbohydrate, high-fat diet-fed rats supplemented with *Sarconema filiforme*.

**Table 8:** Correlation between bacterial community structure and physiological parameters (*p* < 0.05).

Physiological Variables	R^2^	*p*-Value
Epididymal fat	0.59	0.001
Water intake	0.58	0.002
Total abdominal fat	0.56	0.001
Retroperitoneal fat	0.54	0.002
Systolic blood pressure	0.53	0.001
Left ventricle and septum wet weight	0.47	0.001
Fat mass	0.46	0.001
Kidneys wet weight	0.44	0.002
Omental fat	0.40	0.007
Body weight	0.39	0.004
Liver wet weight	0.37	0.011
Right ventricle wet weight	0.33	0.003
Oral glucose tolerance area under the curve	0.33	0.025
Oral glucose tolerance 120-minute blood glucose	0.29	0.027
Food intake	0.25	0.033

**Table 9:** Taxonomic assignments of the zOTUs strongly correlated with physiological parameters.

OTU_ID	Phylum	Family	Genus	Correlation with Physiological Parameters
Zotu42	Actinobacteria	Bifidobacteriaceae	*Bifidobacterium*	Water intake (−)
Zotu20	Bacteroidetes	Bacteroidaceae	*Bacteroides*	Left ventricle and septum wet weight (+), water intake (−)
Zotu1036	Bacteroidetes	Muribaculaceae	unclassified	Water intake (−)
Zotu1144	Bacteroidetes	Muribaculaceae	unclassified	Water intake (−)
Zotu21	Bacteroidetes	Muribaculaceae	unclassified	Water intake (−)
Zotu27	Bacteroidetes	Muribaculaceae	unclassified	Water intake (−)
Zotu541	Bacteroidetes	Muribaculaceae	unclassified	Epididymal fat (+)
Zotu79	Bacteroidetes	Muribaculaceae	unclassified	Water intake (−)
Zotu857	Bacteroidetes	Muribaculaceae	unclassified	Water intake (−)
Zotu10	Bacteroidetes	Prevotellaceae	*Prevotellaceae* UCG-001	Left ventricle and septum wet weight (+), water intake (−)
Zotu77	Firmicutes	Lachnospiraceae	*Anaerostipes*	Food intake (−), water intake (+)
Zotu244	Firmicutes	Lachnospiraceae	*Blautia*	Water intake (+)
Zotu856	Firmicutes	Lachnospiraceae	*Lachnospiraceae* FCS020 group	Water intake (+)
Zotu100	Firmicutes	Lachnospiraceae	*Lachnospiraceae* NK4A136 group	Water intake (−)
Zotu37	Firmicutes	Lachnospiraceae	*Lachnospiraceae* NK4A136 group	Left ventricle and septum wet weight (−)
Zotu762	Firmicutes	Lachnospiraceae	*Lachnospiraceae* NK4A136 group	Water intake (+)
Zotu556	Firmicutes	Lachnospiraceae	*Roseburia*	Food intake (−), water intake (+)
Zotu582	Firmicutes	Lachnospiraceae	*Roseburia*	Food intake (−)
Zotu604	Firmicutes	Lachnospiraceae	*Roseburia*	Food intake (−)
Zotu625	Firmicutes	Lachnospiraceae	*Roseburia*	Water intake (+)
Zotu634	Firmicutes	Lachnospiraceae	*Roseburia*	Food intake (−)
Zotu101	Firmicutes	Lachnospiraceae	unclassified	Water intake (−)
Zotu174	Firmicutes	Lachnospiraceae	unclassified	Food intake (−)
Zotu198	Firmicutes	Lachnospiraceae	unclassified	Left ventricle and septum wet weight (−)
Zotu25	Firmicutes	Lachnospiraceae	unclassified	Water intake (+)
Zotu516	Firmicutes	Lachnospiraceae	unclassified	Water intake (+)
Zotu52	Firmicutes	Lachnospiraceae	unclassified	Left ventricle and septum wet weight (−)
Zotu530	Firmicutes	Lachnospiraceae	unclassified	Water intake (+)
Zotu590	Firmicutes	Lachnospiraceae	unclassified	Left ventricle and septum wet weight (−)
Zotu73	Firmicutes	Lachnospiraceae	unclassified	Water intake (+)
Zotu757	Firmicutes	Lachnospiraceae	unclassified	Food intake (−)
Zotu891	Firmicutes	Lachnospiraceae	unclassified	Water intake (+)
Zotu988	Firmicutes	Lachnospiraceae	unclassified	Water intake (+)
Zotu398	Firmicutes	Peptococcaceae	unclassified	Water intake (−)
Zotu279	Firmicutes	Ruminococcaceae	*Ruminiclostridium*	Left ventricle and septum wet weight (−), water intake (+)
Zotu614	Firmicutes	Ruminococcaceae	*Ruminiclostridium*	Left ventricle and septum wet weight (−)
Zotu643	Firmicutes	Ruminococcaceae	*Ruminiclostridium 9*	Body weight (+), retroperitoneal fat (+), epididymal fat (+), omental fat (+), Total abdominal fat (+), fat mass (+), Liver wet weight (+), Left ventricle and septum wet weight (+), oral glucose tolerance 120-minute blood glucose (+), systolic blood pressure (+), water intake (−)
Zotu133	Firmicutes	Ruminococcaceae	*Ruminiclostridium 9*	Water intake (+)
Zotu135	Firmicutes	Ruminococcaceae	*Ruminiclostridium 9*	Water intake (+)
Zotu63	Firmicutes	Ruminococcaceae	*Ruminiclostridium 9*	Left ventricle and septum wet weight (−)
Zotu595	Firmicutes	Ruminococcaceae	*Ruminococcaceae* UCG-014	Epididymal fat (+), retroperitoneal fat (+), right ventricle wet weight (+), systolic blood pressure (+)
Zotu232	Firmicutes	Ruminococcaceae	*Ruminococcaceae* UCG-014	Liver wet weight (+)
Zotu62	Firmicutes	Ruminococcaceae	unclassified	Left ventricle and septum wet weight (−), water intake (+)
Zotu40	Proteobacteria	Desulfovibrionaceae	*Bilophila*	Epididymal fat (+), omental fat (+), retroperitoneal fat (+), total abdominal fat (+), kidney wet weight (+), liver wet weight (+), oral glucose tolerance 120-minute blood glucose (+), oral glucose tolerance area under the curve (+), systolic blood pressure (+)

Differential abundance analysis was performed using Mvabund. This table includes the physiological parameters strongly correlated *(P* < 0.05) with the bacterial community and incorporates zOTUs that interact with at least 1 of these parameters. Plus sign (+) indicates positive correlations, while minus sign (−) indicates negative correlations.
